# Polygenic scores for complex traits are associated with changes in concentration of circulating lipid species

**DOI:** 10.1371/journal.pbio.3002830

**Published:** 2024-09-26

**Authors:** Rubina Tabassum, Nina Mars, Pietro Della Briotta Parolo, Mathias J. Gerl, Christian Klose, FinnGen, Matti Pirinen, Kai Simons, Elisabeth Widén, Samuli Ripatti

**Affiliations:** 1 Institute for Molecular Medicine Finland, HiLIFE, University of Helsinki, Helsinki, Finland; 2 Broad Institute of the Massachusetts Institute of Technology and Harvard University, Cambridge, Massachusetts, United States of America; 3 Lipotype GmbH, Dresden, Germany; 4 Department of Public Health, Clinicum, Faculty of Medicine, University of Helsinki, Helsinki, Finland; 5 Department of Mathematics and Statistics, University of Helsinki, Helsinki, Finland; Duke University, UNITED STATES OF AMERICA

## Abstract

Understanding perturbations in circulating lipid levels that often occur years or decades before clinical symptoms may enhance our understanding of disease mechanisms and provide novel intervention opportunities. Here, we assessed if polygenic scores (PGSs) for complex traits could detect lipid dysfunctions related to the traits and provide new biological insights. We constructed genome-wide PGSs (approximately 1 million genetic variants) for 50 complex traits in 7,169 Finnish individuals with routine clinical lipid profiles and lipidomics measurements (179 lipid species). We identified 678 associations (*P* < 9.0 × 10^−5^) involving 26 traits and 142 lipids. Most of these associations were also validated with the actual phenotype measurements where available (89.5% of 181 associations where the trait was available), suggesting that these associations represent early signs of physiological changes of the traits. We detected many known relationships (e.g., PGS for body mass index (BMI) and lysophospholipids, PGS for type 2 diabetes and triacyglycerols) and those that suggested potential target for prevention strategies (e.g., PGS for venous thromboembolism and arachidonic acid). We also found association of PGS for favorable adiposity with increased sphingomyelins levels, suggesting a probable role of sphingomyelins in increased risk for certain disease, e.g., venous thromboembolism as reported previously, in favorable adiposity despite its favorable metabolic effect. Altogether, our study provides a comprehensive characterization of lipidomic alterations in genetic predisposition for a wide range of complex traits. The study also demonstrates potential of PGSs for complex traits to capture early, presymptomatic lipid alterations, highlighting its utility in understanding disease mechanisms and early disease detection.

## Abstract 2

Could changes in levels of circulating lipid species be predictive of disease? This study reveals that polygenic scores for a range of complex traits are associated with changes in concentration of circulating lipid species.

## Introduction

Lipids play an important role in vital and diverse metabolic functions such as energy storage and metabolism, signaling, and as structural components and hormones [1]. Dysregulations of the biological pathways or networks involved in these functions, generally under the influence of genetic and environmental factors, may result in increased risk to various complex disorders such as diabetes, cardiovascular disorders, cancers, inflammatory, and neurological disorders [2–4]. Understanding perturbations in circulating lipid levels that often occur years or decades before the presentation of clinical symptoms may enhance our understanding of disease mechanisms and provide novel intervention opportunities.

Complex disorders are generally polygenic in nature and one of the successful ways to predict risk for a complex disorder before clinical symptoms appear is polygenic scores (PGS) approach [5]. PGS, derived from the weighted sum of effects of genetic variants ranging from a few variants to over millions, have emerged as a powerful tool in predicting and classifying disease risk [6]. PGS for coronary artery disease (CAD) have been shown to provide prediction comparable to monogenic mutations of hypercholesterolemia [7]. Though PGS are increasingly becoming accurate and efficient, they represent cumulative effect of large number of genetic variants from complex biological network that need to be dissected for better understanding of disease mechanism.

In light of this, integrating lipidomics that allow simultaneous measurement of hundreds of lipid species [8,9] and PGSs to capture presymptomatic alterations in lipids for a wide range of common disorders provides an unprecedented opportunity to understand physiological changes at molecular level and get insights into the mechanisms. In this direction, Fang and colleagues utilized nuclear magnetic resonance (NMR)-based metabolomics to demonstrate changes in plasma metabolites in increased genetic risk of common diseases in the UK Biobank data [10]. Another recent study by Julkunen and colleagues provided an atlas of associations of NMR-based metabolites to prevalence, incidence, and mortality of common diseases using longitudinal health records in the UK Biobank [11]. However, these studies utilized NMR based metabolites data that include total lipid contents in lipoprotein subclasses but lack information about molecular lipid species. Thus, changes in individual lipid species concentrations associated with increased disease risk before the onset of clinical symptoms remain largely unexplored.

Here, we used genome-wide PGSs to explore relationships between genetic risk for 50 complex traits and plasma lipidome (179 lipid species) in 7,169 participants from the Finnish population. The study aimed to address 2 main questions: (1) do PGSs for complex traits capture underlying lipid dysfunctions? and (2) could PGS-lipid relationships provide new insights to the disease mechanisms? We identified 678 PGS-lipid associations with profound perturbations in plasma lipidome for PGSs for cardiometabolic traits and validated many of these relationships utilizing actual phenotype measurements where available. Altogether, our study provides a comprehensive characterization of lipidomic alterations in genetic risk for a wide range of complex traits. Our results also demonstrate ability of PGSs for complex traits to capture early, presymptomatic lipid alterations, highlighting its potential utility in understanding disease mechanisms and early disease detection.

## Results

### Lipidomic profiles of the GeneRISK cohort

**Fig 1:**
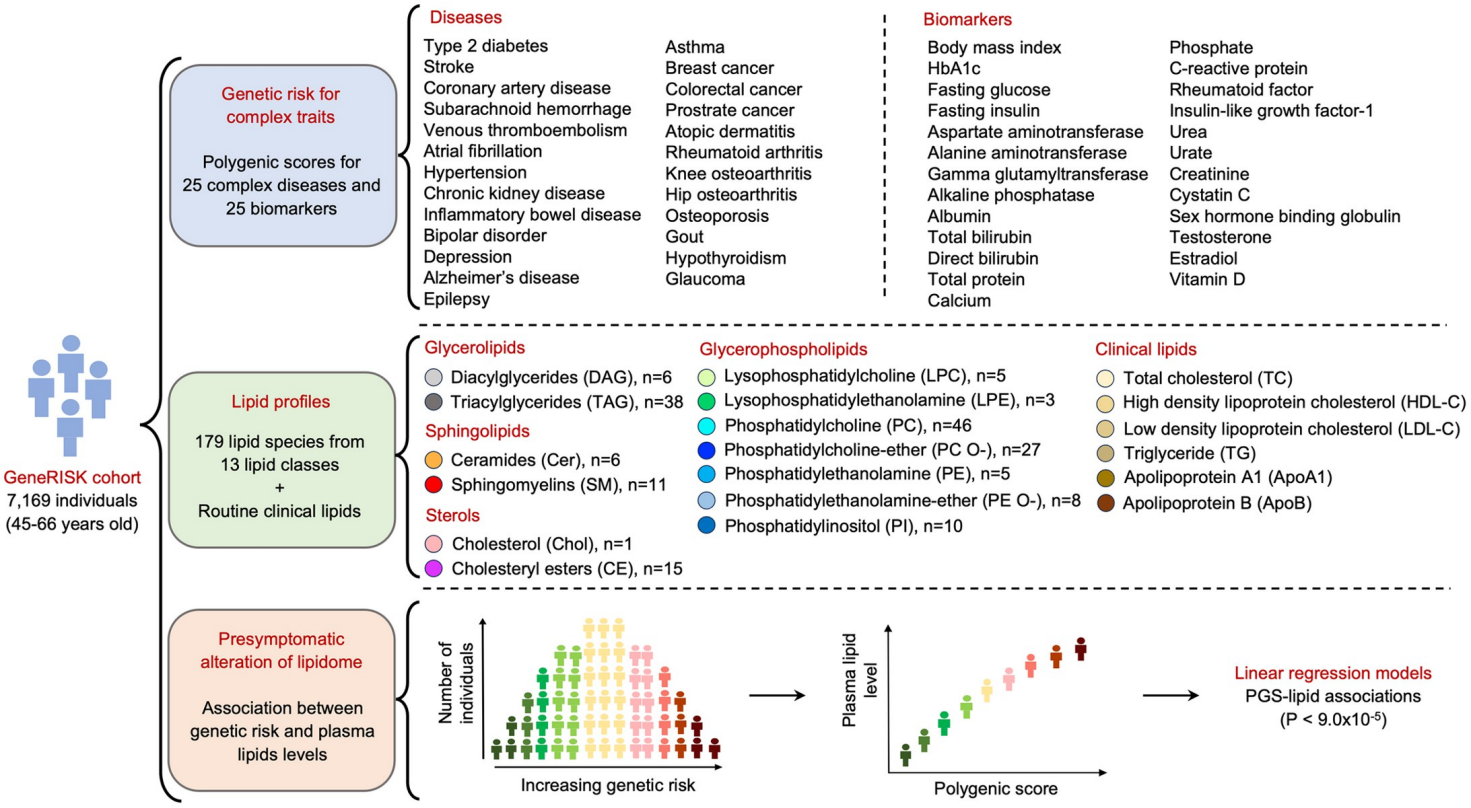
Study design. Overview of the study design and analytical approach, along with the details of the diseases and biomarkers for PGS estimation and lipids included in the study, are illustrated.

The study included 7,169 middle-aged Finnish individuals (mean age ± SD: 55.8 ± 5.8 and 68% women) who participated in the GeneRISK study [12] and had genomics data and measurements for plasma lipidomes and routine clinical lipids. The overall study design and general characteristics of the study participants are summarized in Fig 1 and Table 1, respectively. High-quality lipid species (*N* = 179) measured by shortgun lipidomics were included (S1 Table). Expected relationships between the routine clinical lipids (LDL-C, HDL-C, triglycerides, total cholesterol, apolipoprotein A1, and apolipoprotein B) and lipidomic measurements were detected (S1 Fig and S2 Table). For example, triacylglycerol species (TAGs) were positively correlated with triglycerides, and sterols (cholesteryl esters (CEs) and free cholesterol) were positively correlated with total cholesterol and LDL-C (S1 Fig).

**Table 1:** Basic characteristics of the study cohort.

Characteristics	Women	Men
**N**	4,577	2,592
Age (years)	55.7 (5.7)	55.9 (5.9)
Body mass index (kg/m^2^)	27.2 (5.3)	27.7 (4.4)
Waist circumference (cm)	90.7 (13.4)	100.6 (12.5)
Fasting glucose (mmol/l)	5.61 (0.83)	5.94 (1.0)
Low-density lipoprotein cholesterol (mmol/l)	3.31 (0.94)	3.44 (0.92)
High-density lipoprotein cholesterol (mmol/l)	1.79 (0.50)	1.43 (0.45)
Triglycerides (mmol/l)	1.15 (0.65)	1.49 (1.18)
Total cholesterol (mmol/l)	5.61 (1.04)	5.52 (1.02)
Apolipoprotein A1 (mmol/l)	1.75 (0.29)	1.58 (0.26)
Apolipoprotein B (mmol/l)	0.97 (0.25)	1.06 (0.27)
Systolic blood pressure (mmHg)	126.6 (16.7)	134.7 (15.7)
Diastolic blood pressure (mmHg)	84.3 (9.9)	88.3 (10.0)
Current smokers (N (%))	701 (15.1)	555 (21.2)
Lipid lowering medication (N (%))	432 (9.3)	373 (14.2)
Blood pressure lowering medication (N (%))	948 (20.7)	630 (24.3)

Data are presented as mean (standard deviation) or number (%).

### PGS-lipid associations

**Fig 2:**
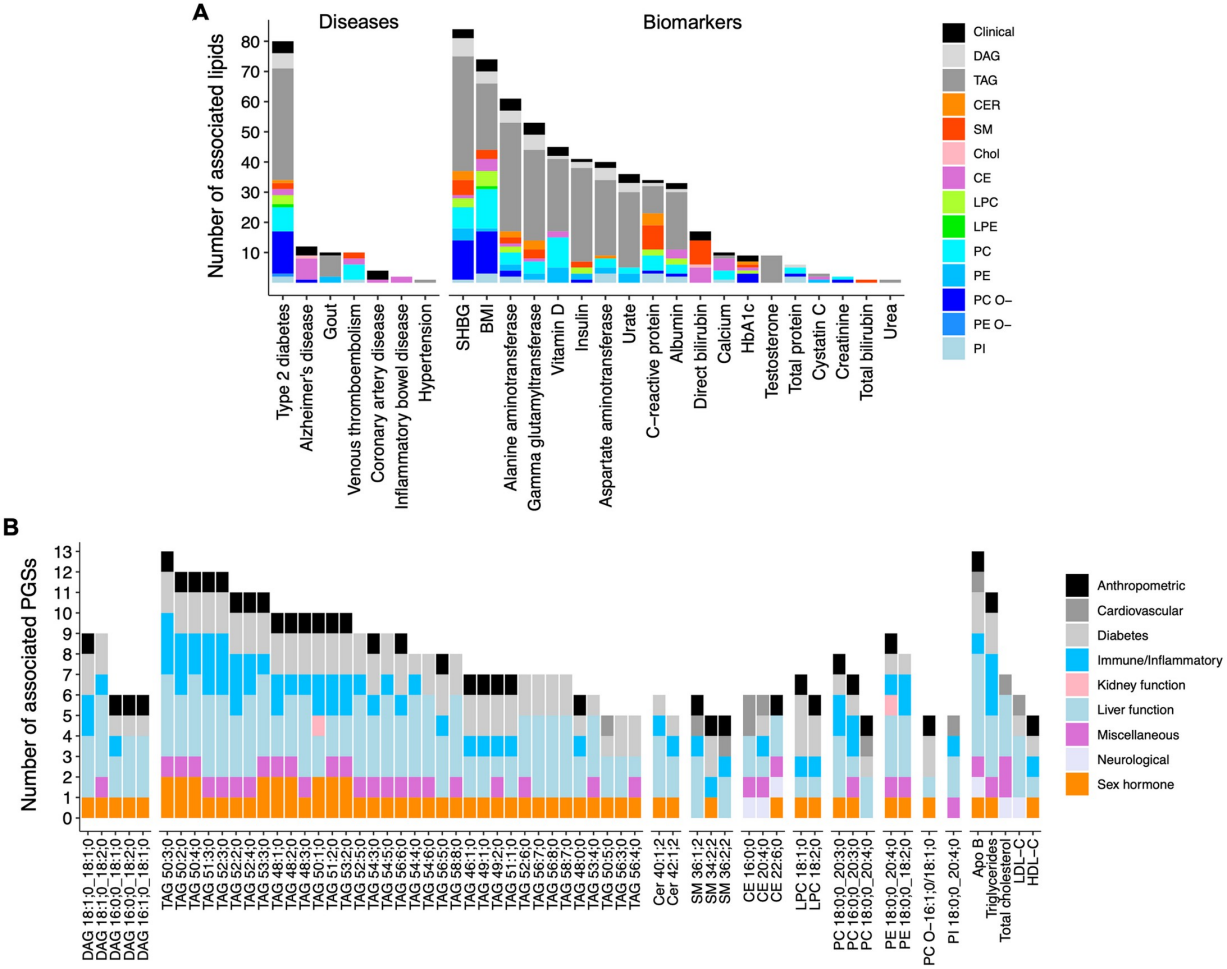
Associations of PGSs with plasma lipidome. (A) Number of lipids associated with PGSs for diseases and biomarkers. The bars are colored based on the lipid classes of the lipid species. (B) Number of PGSs associated with each lipid. The bars are colored based on the PGS category. Only the lipids associated with at least 5 PGSs are shown. BMI, body mass index; Clinical: routine clinical lipid measures; CE, cholesteryl ester; CER, Ceramide; DAG, Diacylglycerol; LPC, lysophosphatidylcholine; LPE, lysophosphatidylamine; PC, phosphatidylcholine; PC O-, phosphatidylcholine-ether; PE, phosphatidylamine; PI, phosphatidylinositol; SM, sphingomyelin; SHBG, sex hormone-binding globulin; TAG, triacylglycerol. The data underlying this figure may be found in S1 Data.

We obtained genome-wide summary statistics for 50 complex traits from 8 disease categories including 25 diseases and 25 biomarkers as detailed in S3 Table and constructed genome-wide PGS for each of these traits in the GeneRISK cohort (Fig 1). Overall correlations between the PGSs were low but hierarchical clustering did capture many expected relationships between the traits, e.g., body mass index (BMI) and C-reactive protein; and BMI and type 2 diabetes (S2 Fig and S4 Table). The association analyses for each of the 50 PGSs across the lipids (179 lipid species and 6 routine clinical lipids) identified 678 PGS-lipid associations that were significant after multiple testing correction (*P* < 9.0 × 10^−5^) (S5 Table). Of the 50 PGSs, PGSs for 7 diseases and 19 biomarkers showed associations with at least one of the lipids (Fig 2A). PGS for type 2 diabetes had association with most lipids (*N* = 80) followed by that of Alzheimer’s disease (*N* = 12), venous thromboembolism (*N* = 10), gout (*N* = 10), CAD (*N* = 4), inflammatory bowel disease (*N* = 2), and hypertension (*N* = 1) (Fig 2A). Among the PGSs for biomarkers, PGS for sex hormone-binding globulin (SHBG) had highest number of associations (84 lipid species), followed by BMI (74 lipid species) and liver function markers (Fig 2A).

Of the 678 PGS-lipid associations, 467 (68.9%) remained significant (*P* < 9.0 × 10^−5^) in the sensitivity analysis performed after excluding 1,222 cases of type 2 diabetes, venous thromboembolism, CAD, gout, inflammatory bowel disease, and Alzheimer’s disease (see “Materials and methods”) (S5 Table). All the 678 PGS-lipid associations were at least nominally significant (*P* < 0.05) in this sensitivity analysis with consistent direction of effects and similar effect sizes (R^2^ = 0.94, *P* < 2.2 × 10^−16^) (S5 Table).

Considered from the lipid’s perspective, TAGs showed the highest number of associations compared with other lipid classes (Fig 2B). Interestingly, phospholipids containing C 20:3;0 and C 20:4;0 polyunsaturated fatty acids (PUFAs) showed more associations than other phospholipids (Fig 2B). To explore this further, we performed an exploratory analysis evaluating association of PGSs with the lipid indices (product-to-precursor ratios) representing fatty acid desaturases and elongases activities in PUFA metabolism (S6 Table), as described previously [13]. This analysis suggested association of PGS for BMI with reduced Δ-5 desaturase (D5D) activity and association of increased Δ-6 desaturase (D6D) activity with PGSs for BMI and type 2 diabetes (S7 Table).

### Comparison between PGS-lipid associations and Pheno-lipid associations

**Fig 3:**
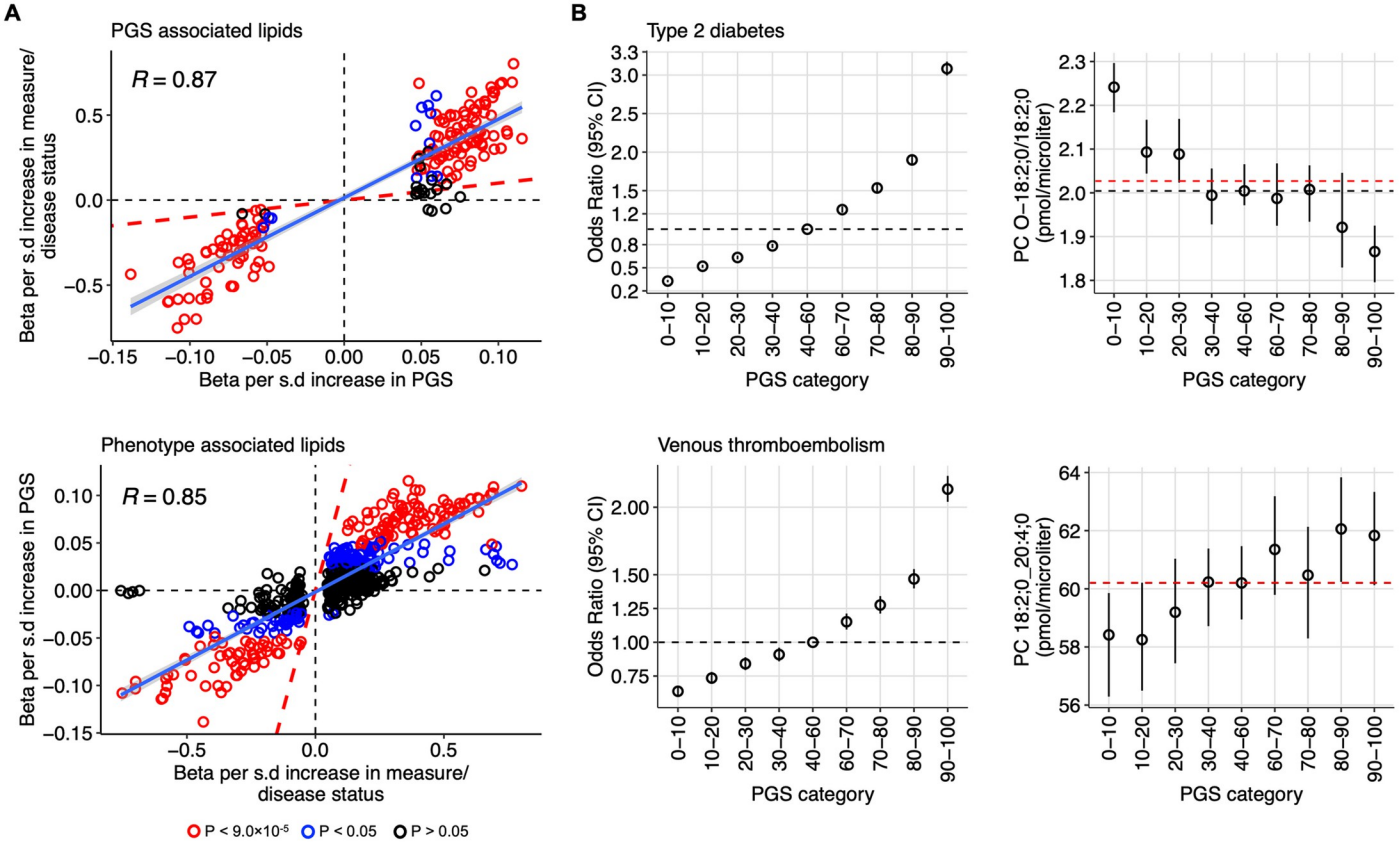
Potential of PGSs for complex traits in capturing early lipid alterations. (A) Validation of PGS-lipid associations using disease status or actual measures of biomarkers. The scatter plot in the upper panel shows the validation of PGS-lipid associations with the actual measures. The x-axis represents the changes (beta in standardized unit) in lipids per standard deviation (SD) increase in the PGS. The y-axis represents the corresponding changes in lipids per SD increase in actual biomarker measures or with disease status. Each dot on the plot represents a lipid with at least one significant PGS-lipid association. The scatter plot in the lower panel shows the comparison of Pheno-lipid associations with the PGS-lipid associations. The x-axis represents the changes (beta in standardized unit) in lipids per standard deviation (SD) increase in actual biomarker measures or with disease status. The y-axis represents the corresponding changes in lipids per s.d. increase in the PGSs. Each dot represents a lipid with at least 1 significant Pheno-lipid association. (B) Exemplar relationship between genetic risk and lipid concentrations. The left side of the upper and lower panels shows the risk of type 2 diabetes and venous thromboembolism respectively, in the FinnGen participants in different percentile categories with 40–60 percentile as reference. The black dotted lines mark the odds ratio of 1 for reference groups (40–60 percentile). The right side shows the median levels of the most strongly associated lipids species for the 2 PGSs-PC O-18:2;0/18:2;0 and PC 18:0;0_20:4;0, respectively, in different percentiles in the GeneRISK cohort. The 95% confidence intervals are shown as error bars. The black dotted lines mark the median levels of the lipids in the reference groups (40–60 percentile), whereas the red dotted lines show the median levels in the full cohort. The data underlying this figure may be found in S1 Data.

To validate the identified PGS-lipid associations, we performed analysis to evaluate lipidomic alterations associated with disease status or actual measures of biomarkers (referred as Pheno-lipid associations) in our cohort where available. Five diseases—type 2 diabetes (N_cases_ = 499), venous thromboembolism (N_cases_ = 232), CAD (N_cases_ = 134), gout (N_cases_ = 37), inflammatory bowel disease (N_cases_ = 438), and 3 biomarkers—BMI, fasting glucose, and systolic blood pressure were included. Of the 181 PGS-lipid associations tested, 162 (89.5%) showed at least nominal Pheno-lipid associations (*P* < 0.05) with the consistent direction of effects and strong correlation between their effect estimates (rho = 0.87) (Fig 3A (upper panel) and S8 Table). The comparisons of the effect sizes for PGS-lipid associations and Pheno-lipid associations for each trait are shown in S3 Fig. To illustrate that PGS-lipid associations represent early signs of physiological changes occurring before clinical symptoms, we have presented 2 exemplar PGS-lipid associations in Fig 3B. The risk of type 2 diabetes and venous thromboembolism in individuals in different percentiles of the respective PGSs, compared to individuals in the 40% to 60% of the PGSs, were determined using data from 438,613 participants from the FinnGen cohort (S9 Table). The Fig 3B shows simultaneous changes in the lipid concentrations with increase in the risk for type 2 diabetes and venous thromboembolism in higher percentiles of PGSs (S10 Table).

Though most of the PGS-lipid associations were validated in Pheno-lipid associations, PGSs captured only 51.9% (283/545) of the Pheno-lipid associations at nominal significance (*P* < 0.05) (Fig 3A (lower panel) and S8 Table). Fasting plasma glucose level was associated with 134 lipids but none of these were observed as significant PGS-lipid associations. Similarly, many of the lipid species associated with the measured BMI were not associated with the PGS for BMI. Also, the effect estimates were higher for Pheno-lipid associations compared with that of the PGS-lipid associations (Fig 3A). Though the statistical power and efficiency of the PGSs could not be ruled out for these differences, the Pheno-lipid associations not captured by PGSs might reflect the consequences of the phenotypes and might help to distinguish risk factors from the effects of the phenotypes.

### Lipidomic signatures of PGSs for complex diseases

**Fig 4:**
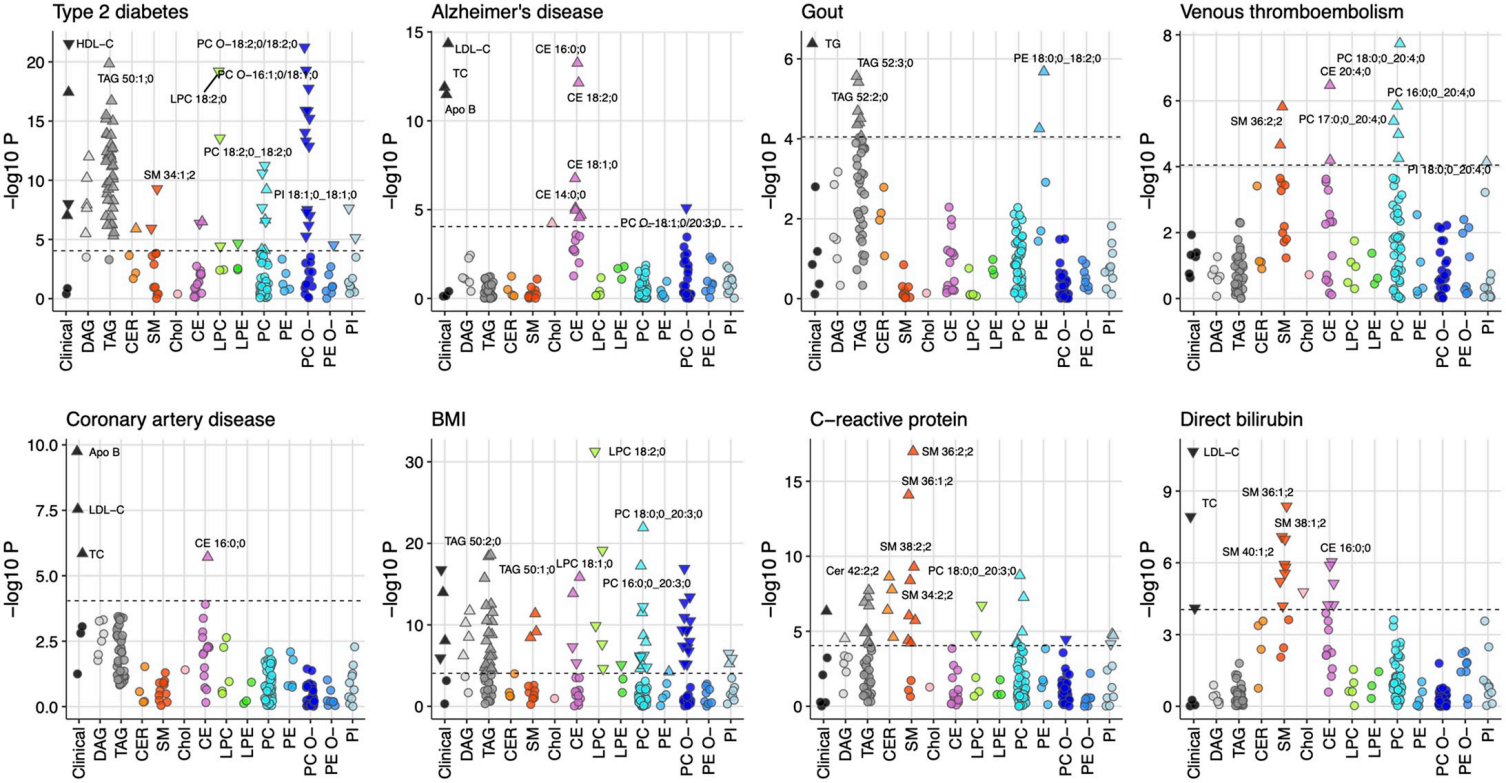
Lipidomic signatures of PGSs for complex traits. The Manhattan plots show the associations of selected PGSs with lipids. The lipids are grouped and colored by the lipid classes they belong. Upright triangle denotes positive effect on lipid and upside-down triangle represent negative effect. The dotted horizontal line represents the threshold for significant associations at *P* < 9.0 × 10^−5^. BMI, body mass index; CE, cholesteryl ester; CER, ceramide; Chol, free cholesterol; DAG, diacylglycerol; LPC, lysophosphatidylcholine; LPE, lysophosphatidylamine; PC, phosphatidylcholine; PC O-, phosphatidylcholine-ether; PE, phosphatidylamine; PE O-, phosphatidylamine-ether; PI, phosphatidylinositol; SM, sphingomyelin; TAG, triacylglycerol. The data underlying this figure may be found in S1 Data.

The effects of many of the PGSs on lipidome were found to be correlated, despite low correlations between the PGSs themselves. For example, strong correlations were found between the effect estimates for PGSs for type 2 diabetes and SHBG (rho = −0.91), C-reactive protein and BMI (rho = 0.85), gout and SHBG (rho = −0.86) (S4 Fig and S11 Table). Thus, examining PGS-lipid association patterns across PGSs may help to understand common mechanisms of lipidomic changes between the traits and to identify molecular differences in similar traits. The PGS-lipid associations for the selected PGSs are shown in Fig 4. Below we present findings for 2 PGSs—type 2 diabetes and venous thromboembolism, that provide important biological insights.

#### Type 2 diabetes

**Fig 5:**
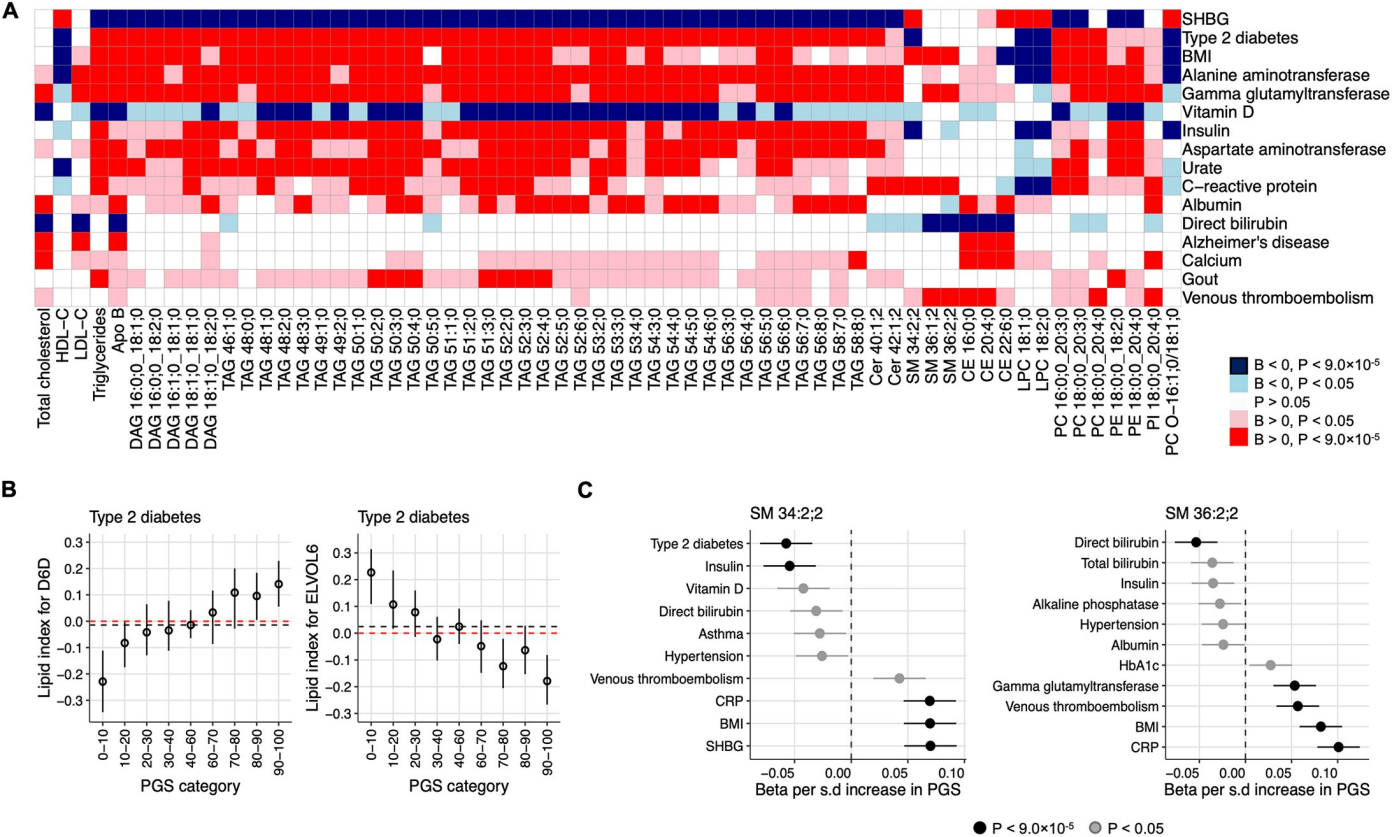
Sharing and differences in the lipidomic alterations in genetic risks across the traits. **(A)** The heatmap shows the pattern of associations of lipids across the PGSs. The light blue shade represents negative effect (beta <0) with *P* < 0.05, dark blue represents negative effect with *P* < 9.0 × 10^−5^. The pink shade represents positive effect (beta >0) with *P* < 0.05, red represents positive effect with *P* < 9.0 × 10^−5^. White shade denotes no association (*P* > 0.05). Only the PGSs and lipids with at least 10 and 5 significant associations (*P* < 9.0 × 10^−5^), respectively, are shown. BMI, body mass index; SHBG, sex hormone-binding globulin. (B) Association of PGS for type 2 diabetes with lipid indices suggesting altered D6D and ELVOL6 activities. The median levels of the lipid indices representing D6D and ELVOL6 activities are plotted for individuals in different percentiles in the GeneRISK cohort. The 95% confidence intervals are shown as error bars. The black dotted lines mark the median levels of the lipids in the reference groups (40–60 percentile), whereas the red dotted lines show the median levels in full cohort. (C) Association of SM 34:2;2 and SM 36:2;2 with the PGSs. The change (beta in standardized unit) in lipids per standard deviation (SD) increase in PGSs with 95% confidence intervals are shown. The data underlying this figure may be found in S1 Data.

PGS for type 2 diabetes had a profound effect on plasma lipidome with strong associations with the increase in most of the glycerolipids (diacylglycerols (DAGs) and TAGs) (Fig 5A). Consistently, PGS for type 2 diabetes showed association with routine triglyceride measure and HDL-C, however, no significant association was found either with LDL-C or total cholesterol (Fig 5A). Interestingly, we observed that phosphatidylcholines (PCs) with C18:2 were negatively associated PGS for type 2 diabetes, whereas PCs with C 20:3 were positively associated (S5 Table). Further exploration in association analyses with lipid indices for fatty acid desaturases and elongases found association of PGS for type 2 diabetes with increased D6D and decreased ELOVL fatty acid elongase 6 (ELOVL6) activities (Fig 5B and S7 Table).

PGS for type 2 diabetes seemed to have a similar lipidomic alterations with PGSs for other traits including BMI, SHBG, liver function markers, C-reactive protein, and fasting insulin (Fig 5A). Consistently, strong correlations between their effect estimates were found (S4 Fig and S11 Table), suggesting probable common mechanisms of lipid dysfunctions in genetic risk for these traits. For example, similar to PGS for type 2 diabetes, altered activities of ELOVL6 and D6D in the high PGSs for BMI and SHBG were also suggested (S7 Table). However, we also observed some differences between the associations of these traits with lipids. Many of the long polyunsaturated TAGs associated with PGS for type 2 diabetes were not associated with the PGS for BMI, even though these TAGs were associated with the actual measure of BMI (Fig 5A). This suggest that while these lipids might be on a causal pathway or risk factors for type 2 diabetes, they could represent a consequence of a part of high BMI without a strong genetic component. Similarly, we observed differences in the effect estimates of PGSs for diabetes related traits—type 2 diabetes, fasting glucose, and HbA1c. Unlike PGS for type 2 diabetes that associated with many lipid species with large impact on TAGs and PC O- species, PGS for fasting glucose did not show any significant association but had heterogeneity in the effect sizes for 75 of 80 lipids associated with type 2 diabetes (P_het_ <0.05) (S12 Table). Moreover, there were differences in the effects of PGSs for type 2 diabetes and HbA1c for 71 out of 84 lipid species associated with either of the PGSs (P_het_ <0.05) (S13 Table).

Another interesting observation while comparing the associations of PGSs across the traits was their relationships with SMs. While majority of the lipids had consistent direction of effect across the PGSs, SM 34:2;2 had effects in opposite directions for PGSs. SM 34:2;2 was positively associated with PGSs for BMI and CRP but was negatively associated with PGSs for type 2 diabetes and fasting insulin (Fig 5C). This inverse relationship for SM 34:2;2 was also observed in the Pheno-lipid associations for BMI and type 2 diabetes.

#### Venous thromboembolism

PGS for venous thromboembolism showed associations with increased levels of 10 lipids including 6 lipids (5 PCs and 1 CE) containing C 20:4 as a fatty acid chain (Figs 4 and 5), reflecting a potential contribution of C 20:4 fatty acid to the risk of venous thromboembolism. To test this, we performed association of PGS for venous thromboembolism with fatty acid aggregates across all lipid classes (e.g., C 16:0;0) and fatty acid aggregates per lipid class (e.g., PC 16:0;0) that were calculated as sum of the molar concentration of fatty residues within a lipid class. The association analysis with fatty acid aggregates showed that PGS for venous thromboembolism was associated with C 20:4 fatty acid (S5 Fig), as has been suggested previously [14]. However, we did not find any evidence for its association with altered activity of enzymes involved in PUFA metabolism (S7 Table).

Comparing the lipidomic signature of PGS for venous thromboembolism across the traits, we found that a sphingomyelin (SM) species—SM 36:2;2 associated with PGSs for venous thromboembolism was also associated with PGSs for BMI, C-reactive protein, direct bilirubin, and gamma-glutamyl transferase (Fig 5A and S5 Table), suggesting a probable common link between these traits. A causal relationship between venous thromboembolism risk and favorable adiposity has been suggested previously despite the protective metabolic effect of favorable adiposity [15]. We further explored this by examining the association of PGS for favorable adiposity with the lipidome in our cohort (see Material and methods). The PGS for favorable adiposity was associated with decreased TAGs and ceramides (CERs) consistent with its protective metabolic effect but with increased SMs including SM 36:2;2 (S14 Table), suggesting that the increased risk of venous thromboembolism in favorable obesity may be mediated through SMs.

## Discussion

Lipidomes combine signals from multiple origins into a systemic readout and may provide unique insights needed for innovative medical interventions. Utilizing the lipidomics- and genomics-based approaches, we present a catalogue of alterations in plasma lipidome associated with the genetic risk of complex diseases or genetically determined higher levels of clinical biomarkers. Our results revealed profound perturbations in plasma lipidome for many PGSs, particularly for cardiometabolic traits and validated many of these relationships utilizing actual phenotype measurements. Our results substantiate many trait—lipid and trait—trait relationships with strong literature support and provide new biological insights, as discussed below.

First, our results demonstrate potential of PGSs for complex traits in capturing physiological changes related to complex traits at molecular level. PGSs for complex diseases are becoming increasing popular and efficient in predicting disease risk, however, their effects at molecular levels are less explored. Our study presents the first report, to the best of our knowledge, of presymptomatic alterations in molecular lipid species examined simultaneously for genetic risk of a wide range of complex traits. These findings are supported by 3 important observations in our study—(1) validation of PGS-lipid associations after excluding the disease cases; (2) concordance between the associations of PGSs and actual measures with the lipidome; and (3) detection of disease—lipid associations with strong literature support. The results from the association analysis after excluding known disease cases suggested that the identified PGS-lipid associations are largely contributed by the alterations in relatively healthy or undiagnosed individuals. We further show that most of the alterations associated with PGSs were also associated with the actual phenotype measure, suggesting that the identified PGS-lipid associations represent early signs of physiological changes occurring before clinical symptoms.

Detection of many well-recognized associations between the traits and lipids in both PGS-lipid and Pheno-lipid associations also demonstrates the potential of PGSs for complex traits in capturing lipid alterations. We confirmed previously demonstrated associations of BMI with the reduced levels of lysophosphatidylcholines (LPCs) and ether lipids and increased levels of TAGs, DAGs, and SMs [16–18]. The observed strong associations of PGS for type 2 diabetes with DAGs and TAGs agree with the previous reports [19,20]. Also, the association results of PGS for type 2 diabetes with routine clinical lipids are consistent with the recent reports suggesting causal effect of genetically predicted type 2 diabetes risk on TG and HDL-C but no evidence of causal effect on LDL-C and total cholesterol [21,22].

The PGS-lipid associations were consistent with the trait—trait relationships with strong literature support. For example, PGSs for SHBG and type 2 diabetes showed strong negative correlation between their lipidomic profiles. This is consistent with the previous reports from prospective studies that have shown inverse relationship between circulating SHBG levels and risk of type 2 diabetes [23–25]. Circulating SHBG levels have also been linked with metabolic perturbations related to insulin resistance and type 2 diabetes including lipid levels [23]. On the similar lines, we also observed association of perturbations in lipidomic profiles with PGS for testosterone levels, with similar profile as PGSs for SHBG and type 2 diabetes, which is consistent with previous prospective studies [26–28].

Second, our results suggest avenues for prevention strategies. We found that individuals with high genetic risk for a disease harbor risk-associated lipidomic signature of the disease even before the development of clinical symptoms. For example, we found that PGS for venous thromboembolism was predominantly associated with the increased levels of lipids containing arachidonic acid (C 20:4), an n-6 PUFA, suggesting a probable role of arachidonic acid-related pathways in the pathophysiology of venous thromboembolism. A previous mendelian randomization study also suggested a causal effect of arachidonic acid on venous thromboembolism [14]. Thus, our study suggests that strategies to reduce level of circulating arachidonic acid may help in reducing the risk of venous thromboembolism.

Third, our study provided noncanonical associations and new insights to the disease mechanisms. Obesity is a significant risk factor for many complex disorders including type 2 diabetes and cardiovascular diseases, however, not all individuals with obesity develop comorbidities. Several studies have suggested that there could be 2 types of obesity associated genetic variants—those associated with obesity with unfavorable metabolic effect (referred to as unfavorable adiposity), and others associated with obesity with favorable metabolic effect (referred to as favorable adiposity) [15,29,30]. A study by Martin and colleagues has shown causal metabolic effect of favorable adiposity on reduced risk of many diseases including type 2 diabetes, systolic blood pressure, and heart disease [15]. Martin and colleagues also showed that despite favorable metabolic effects like higher HDL-C and lower triglycerides, favorable adiposity could increase risk for certain disorders through non-metabolic effects including venous thromboembolism [15]. In our study, we found that the PGS obtained from the genetic variants associated with favorable adiposity were associated with decreased levels of TAGs, CEs, and PCs but with the increased levels of SMs including SM 36:2;2 (S14 Table). The higher levels of SM 36:2;2 was also associated with increased genetic risk of venous thromboembolism in our study (Fig 5C). These observations point to the possibility that the increased risk of venous thromboembolism in adiposity with favorable metabolic effects could be mediated through the increased levels of SMs; however, further studies are needed to evaluate the effect of SMs on venous thromboembolism. These results also suggest a potential mechanism through which favorable adiposity might increase risk for certain diseases, despite favorable metabolic effect.

Though the study is based on a large population-based cohort with a broad lipidome coverage, it is not without limitations. We acknowledge that PGS provide an estimate of a disease risk that depends on the robustness of its GWAS. Consequently, we may not be able to statistically detect all true associations between genetic component of the trait and a lipid species in cases where those associations are weak compared to the predictive power of the existing PGS. Also, the PGSs were derived based on the GWAS summary statistics from the European ancestry samples and evaluated in the Finnish population. Though high transferability of the PGSs across European populations including Finnish population has been demonstrated [31], possibility of variability in the results could not be ruled out. Also, as suggested in our previous study [32], demographic and other cohort characteristics could affect the lipidome profiles. Thus, it is not clear if findings of this study could be generalized to other populations. Furthermore, lipidomic profiles were measured in whole plasma, which does not provide information at the level of individual lipoprotein subclasses and limits our ability to gain detailed mechanistic insights. Further advances in lipidomics platforms might help to capture more comprehensive and complete lipidomic profiles, including the position of fatty acyl-chains in the glycerol backbone of TAGs and glycerophospholipids and detection of sphingosine-1-P species and several other species, that would allow to overcome these limitations.

In conclusion, our study provides a comprehensive view of lipidomic perturbations under the effect of genetic liability for various complex traits. The study not only corroborated the existing and known relationships between the lipids and complex traits but also provided new and more refined insights by utilizing high-resolution lipidomic profiles. Thus, our study demonstrates the potential of PGS to capture early, presymptomatic lipid alterations, highlighting its potential utility in understanding disease mechanisms and possibly in early disease detection.

## Materials and methods

### Study participants

The study included participants from the following cohorts:

**GeneRISK:** The study included middle-aged participants (aged 45 to 66 years) from the ongoing prospective GeneRISK cohort recruited during 2015–2017 from Southern Finland. The recruitment process and sample collection procedures are described in detail in [12]. Briefly, participants were instructed to fast overnight for 10 h before the blood samples were collected for plasma, serum, and DNA extraction. Fasting serum lipids including HDL-C, LDL-C, triglyerides, total cholesterol, Apolipoprotein A1 and Apolipoprotein B were measured using standard enzymatic methods. GeneRISK study participants’ DNA, blood, serum, and plasma samples, in addition to their demographic information and health data have been stored in the THL Biobank (https://www.thl.fi/en/web/thlfien/topics/information-packages/thl-biobank). The study was carried out according to the principles of the Helsinki declaration and the Council of Europe’s (COE) Convention of Human Rights and Biomedicine. All study participants gave their written informed consent to participate in the study. The study protocols were approved by The Hospital District of Helsinki and Uusimaa Coordinating Ethics committees (approval No. 281/13/03/00/14).
**FinnGen:** This study uses FinnGen study Data Freeze 11 including 438,613 adults (age ≥18) from epidemiological cohorts, disease-based cohorts, and hospital biobanks [www.finngen.fi]. The definition for disease endpoints used in this study—type 2 diabetes and venous thromboembolism, are provided in S9 Table. Study subjects in FinnGen provided informed consent for biobank research, based on the Finnish Biobank Act. Alternatively, separate research cohorts, collected prior the Finnish Biobank Act came into effect (in September 2013) and start of FinnGen (August 2017), were collected based on study-specific consents and later transferred to the Finnish biobanks after approval by Fimea (Finnish Medicines Agency), the National Supervisory Authority for Welfare and Health. Recruitment protocols followed the biobank protocols approved by Fimea. The Coordinating Ethics Committee of the Hospital District of Helsinki and Uusimaa (HUS) statement number for the FinnGen study is Nr HUS/990/2017. Further details on the study permits and ethics committee approval are provided in S1 Text.

### Lipidomics

Lipidomic measurements were performed for 7,292 participants from the GeneRISK cohort using mass spectrometry-based shotgun lipidomic analysis at Lipotype GmbH (Dresden, Germany) as described by Surma and colleagues [33]. For lipid extraction, an equivalent of 1 μl of undiluted plasma was used, and plasma lipids were extracted with methyl tert-butyl ether/methanol (7:2, V:V) [34]. Samples were analyzed by direct infusion in a QExactive mass spectrometer (Thermo Scientific, Bremen, Germany) equipped with a TriVersa NanoMate ion source (Advion Biosciences, Ithaca, New York, United States of America). Samples were analyzed in both positive and negative ion modes with a resolution of *R*_m/z = 200_ = 280,000 for MS and *R*_m/z = 200_ = 17,500 for MSMS experiments in a single acquisition. The Lipidomics Standard Initiative minimal reporting checklist [35] for this study can be found at https://doi.org/10.5281/zenodo.11389457.

#### Lipid nomenclature

Lipid molecules are identified as species or subspecies. Fragmentation of the lipid molecules in MSMS mode delivers subspecies information, i.e., the exact acyl chain (e.g., fatty acid) composition of the lipid molecule. MS only mode, acquiring data without fragmentation, cannot deliver this information and provides species information only. In that case, the sum of the carbon atoms and double bonds in the hydrocarbon moieties is provided. Lipid species are annotated according to their molecular composition as lipid class <sum of carbon atoms>:<sum of double bonds>;< sum of hydroxyl groups>. For example, PI 34:1;0 denotes phosphatidylinositol with a total length of its fatty acids equal to 34 carbon atoms, total number of double bonds in its fatty acids equal to 1 and 0 hydroxylation. In case of sphingolipids, SM 34:1;2 denotes a sphingomyelin species with a total of 34 carbon atoms, 1 double bond, and 2 hydroxyl groups in the ceramide backbone. Lipid subspecies annotation contains additional information on the exact identity of their acyl moieties and their *sn*-position (if available). For example, PI 18:1;0_16:0;0 denotes phosphatidylinositol with octadecenoic (18:1;0) and hexadecanoic (16:0;0) fatty acids, for which the exact position (*sn*-1 or *sn*-2) in relation to the glycerol backbone cannot be discriminated (underline “_” separating the acyl chains). On contrary, PC O-18:1;0/16:0;0 denotes an ether-phosphatidylcholine, in which an alkyl chain with 18 carbon atoms and 1 double bond (O-18:1;0) is ether-bound to *sn*-1 position of the glycerol and a hexadecanoic acid (16:0;0) is connect via an ester bond to the *sn*-2 position of the glycerol (slash “/” separating the chains signifies that the *sn*-position on the glycerol can be resolved). Lipid identifiers of the SwissLipids database (http://www.swisslipids.org) are provided in S1 Table.

#### Postprocessing

Data were analyzed using LipotypeXplorer, a proprietary software developed by Lipotype GmbH, which is based on LipidXplorer [36,37]. Lipids with signal-to-noise ratio >5 and amounts >5-fold higher than in corresponding blank samples were considered. Reproducibility was assessed by the inclusion of 8 reference plasma samples per 96-well plate. Using 8 reference samples per 96-well plate batch, lipid amounts were corrected for batch variations and for analytical drift if the *p*-value of the slope was below 0.05 with R^2^ greater than 0.75 and the relative drift was above 5%. Samples with very low total lipids content and number of lipids detected were removed and lipid species detected in <70% of the remaining samples were also excluded. Furthermore, samples with >30% missingness for the QC passed lipid species were also excluded. After quality control (QC), lipidomics data comprised of 7,266 individuals and 179 lipid species from 13 lipid classes. Median coefficient of variation (%CV) of the reference plasma samples was 12.1% with 80% of the lipid species were measured with a %CV < 20%. Median plasma concentration of each of the lipid species and their %CV are provided in S1 Table.

### Product-to-precursor ratios

Fatty acid desaturases and elongases activities were estimated using product-to-precursor ratios (referred as lipid indices) of sums of fatty acids in all lipids measured on the subspecies level (CE, DAG, TAG, LPC, LPE, PC, PC O−, PE, PE O−, PI) as described previously [13]. The ratio of C 20:4;0 to C 20:3;0 was used to estimate Δ-5-desaturase (D5D) activity and the ratio of C 18:3;0 to C 18:2;0 for D6D activity was used. The ratio of C 20:3;0 to C 18:3;0 for ELOVL5 activity and the ratio of C 18:0;0 to C 16:0;0 for ELOVL6 activity were used. Fatty acid aggregates were calculated as sum of all the lipids containing the fatty acid in a particular lipid class. More details about the calculation of lipid indices and fatty acid aggregates are provided in S6 Table.

### Genotyping and imputation

Genotyping for the GeneRISK study participants was performed using the HumanCoreExome BeadChip (Illumina, San Diego, California, USA). The genotypes were called using GenomeStudio and zCall at the Institute for Molecular Medicine Finland (FIMM). Genotyping data was lifted over to build version 38 (GRCh38/hg38) (as described in dx.doi.org/10.17504/protocols.io.nqtddwn). Pre-imputation QC included exclusion of individuals with <95% call rate, discrepancies between biological and reported sex, extreme heterozygosity (±4 SD), and non-Finnish ancestry as well as of variants with <98% call rate, deviation from Hardy—Weinberg equilibrium (HWE *P* < 1 × 10^−6^) and minor allele frequency (MAF) <0.05. Pre-phasing of genotyped data was performed with Eagle 2.3.5 with the number of conditioning haplotypes set to 20,000 [38]. Imputation was done with Beagle 4.1 [39] (as described in https://doi.org/10.17504/protocols.io.nmndc5e) using population-specific Sequencing Initiative Suomi (SISu) v3 reference panel developed from high-coverage (25 to 30×) whole-genome sequences for 3,775 Finnish individuals. After the quality control, imputed genotype data was available for 7,169 individuals that was used for the calculation of the PGS and subsequent analyses. Genotyping and imputation for the FinnGen cohort has been described previously [40] and are detailed in the S1 Text.

### PGS calculation

The study included 25 diseases including cardiometabolic disorders, cancers, kidney disease, inflammatory/immune, and neurological disorders, and 25 clinical biomarkers for anthropometric traits, glycemic index, liver function, kidney function, and sex hormones (S1 Table). The diseases were chosen based on their potential link with lipids and availability of large, published genome-wide association studies (GWASs) with full summary statistics available for genome-wide PGSs. Further, biomarkers were selected based on their clinical utility in the selected disease prediction or diagnosis and availability of their measurements in the UKBB. For each of these selected diseases and biomarkers, genome-wide PGSs were calculated either using the largest publicly available summary statistics or the summary statistics from the internally run GWAS in the UK Biobank participants, except for insulin, as detailed in S3 Table. PRS-CS was used for inferring posterior effect sizes (weights) for calculation of genome-wide PGSs with 1000 Genomes Project European sample (*N* = 503) as external linkage disequilibrium (LD) reference panel [41]. For insulin, weights were obtained from the PGS catalog (https://www.pgscatalog.org/score/PGS001351/). Using the weights for each disease and biomarker, PGSs were calculated for the GeneRISK participants using linear allele scoring implemented in PLINK2.0 [42].

PGS for favorable adiposity was calculated as described in Martin and colleagues [29]. Martin and colleagues identified 36 variants that were associated with body fat percentage and a composite of favorable metabolic phenotype consisting of higher HDL-C and SHBG, and lower triglycerides and liver enzymes [28]. Out of these 36 variants, 31 were available in our data set. Using the effect sizes of these variants on body fat percentage from the Martin and colleagues’ study [31], PGS for favorable adiposity were calculated for GeneRISK participants using linear allele scoring implemented in PLINK2.0 [42].

### Statistical analyses

The statistical analyses and data visualization were done using R 4.4.0. PGSs were scaled to mean zero and standard deviation of one. For lipid levels, residuals obtained after regressing on age, age^2^, gender, age*gender, collection site, lipid medication, and the first 10 principal components for genetic structure from log transformed lipid levels were normalized using the rank based inverse normal transformation. Association between the PGSs and lipid levels were determined using linear regression models with transformed lipid levels as the dependent variables. Association analyses involving PGSs for breast cancer and estradiol were performed only in women, while that for prostate cancer and testosterone were performed only in men in sex-specific analysis. Sex-specific inverse normal transformation after adjustments for covariates was performed for sex-specific analysis. Associations between PGSs and diseases’ risk for type 2 diabetes and venous thromboembolism in the FinnGen cohort were determined using logistic regression models adjusting for birth year, sex, genotyping array, cohort, and the first 10 principal components for genetic structure. After applying the Bonferroni correction to adjust for multiple testing, results with *P* < 9.0 × 10^−5^ (*P* < 0.05/(70*8) to adjust for 70 PCs that were needed to explain >90% of the overall variation in lipidome and 8 disease categories) were considered statistically significant.

Heterogeneity in the effect sizes between 2 PGSs was estimated using the following equation:

$${\text{Het}}_{ß}={\left({ß}_{\text{a}}–{ß}_{\text{b}}\right)}^{2}/\left({{\text{SE}}_{\text{a}}}^{2}+{{\text{SE}}_{\text{b}}}^{2}\right).$$

The ß and SE represent effect size and standard error obtained from the linear regression model described above. *P*-values for heterogeneity (P_het_) were obtained from Het_ß_ under the null assumption of equal effect sizes in the 2 PGSs (referred as a and b in the equation), from the standard chi-square distribution with 1 degree of freedom.

### Sensitivity analysis

To evaluate the effect of disease status on the identified PGS-lipid associations, we performed sensitivity analysis after excluding disease cases. The disease status for type 2 diabetes, venous thromboembolism, CAD, gout, inflammatory bowel disease, and Alzheimer’s disease was obtained using the nationwide healthcare registries. The disease definitions used to identify disease cases are provided in S15 Table. Additionally, self-reported cases of type 2 diabetes were also excluded. Cases for these diseases were excluded because they showed significant PGS-lipid associations.

## Supporting information

S1 Text Supplementary materials and methods. (DOCX)

S1 Table Details of lipid species included in the study after quality control filtering. (XLSX)

S2 Table Correlations between the lipids. The coefficients for pairwise Spearman’s rank correlation (rho) between the lipids are provided.
(XLSX)

S3 Table Details of GWAS summary statistics used to calculate the PGSs in this study. (XLSX)

S4 Table Correlations between the PGSs for the complex traits. The coefficients for pairwise Spearman’s rank correlation (rho) between the polygenic scores are provided.
(XLSX)

S5 Table Association of PGSs for 50 complex traits with the lipidome including 179 lipid species and 6 routine clinical lipids. (XLSX)

S6 Table Details of lipid indices used in the study. (XLSX)

S7 Table Association of PGSs with the lipid indices. (XLSX)

S8 Table Validation of PGS-lipid associations in the Pheno-lipid association analysis. (XLSX)

S9 Table Disease definitions in FinnGen. All registries are nationwide and contain information on all Finnish citizens.
(XLSX)

S10 Table Association of PGSs for type 2 diabetes and venous thromboembolism with the risk of developing type 2 diabetes and venous thromboembolism respectively. The analysis was performed in the FinnGen participants.
(XLSX)

S11 Table Correlations between the effect of PGSs on the lipidome. Only the traits with at least 10 PGS-lipid associations are included.
(XLSX)

S12 Table Heterogeneity between the effects of PGSs for type 2 diabetes and fasting glucose on lipids. (XLSX)

S13 Table Heterogeneity between the effects of PGSs for type 2 diabetes and HbA1c on lipids. (XLSX)

S14 Table Association of PGS for favorable obesity with plasma lipidome. (XLSX)

S15 Table Disease definitions in GeneRISK cohort. (XLSX)

S1 Fig Correlations between the routine clinical lipid measures and molecular lipid species. Each point in the plots represents the Spearman correlation coefficient (rho). The lipid species are grouped and colored by the lipid classes they belong. The data underlying this figure may be found in S1 Data.
(TIFF)

S2 Fig Correlations between the polygenic scores (PGS). Pair-wise Spearman correlation between the PGS for all 50 complex traits included in the study are shown in the heatmap. The data underlying this figure may be found in S1 Data.
(TIFF)

S3 Fig Comparison of associations of lipidome with measured phenotypes and genetically predicted phenotype. Scatter plots show correlations between the effect sizes (beta per SD increase in PGS) obtained from the linear regression analyses for association of the PGS with the lipids on x-axis and the corresponding effect sizes for the association of actual measure or disease status with the lipids on y-axis. R represents the Spearman correlation coefficient. The red line represents the regression line with slope of one and intercept zero, while blue line represents the regression line of the model. The data underlying this figure may be found in S1 Data.
(TIFF)

S4 Fig Correlation between the effects of the PGSs on the lipidome. Pair-wise Spearman correlation between the effect sizes of each pair of PGSs for lipid species are shown in the heatmap. The z-scores (beta/SD) were used to calculate the correlations between the PGSs. Only the PGSs with at least 10 significant PGS-lipid associations are plotted. The data underlying this figure may be found in S1 Data.
(TIFF)

S5 Fig Association of PGS for venous thromboembolism with fatty acids aggregates. Fatty acid aggregates were calculated across all lipid classes (e.g., C 16:0;0), whereas fatty acid aggregates per lipid class were determined as the sum of the molar concentrations of fatty residues within each lipid class (e.g., PC 16:0;0). Only the lipids with *P* < 0.05 are plotted. The data underlying this figure may be found in S1 Data.
(TIFF)

S1 Data All numerical data underlying the main and supplementary figures. (XLSX)

## Abbreviations

BMI: body mass index
CAD: coronary artery disease
CE: cholesteryl ester
CER: ceramide
GWAS: genome-wide association study
HWE: Hardy–Weinberg equilibrium
LD: linkage disequilibrium
LPC: lysophosphatidylcholine
MAF: minor allele frequency
NMR: nuclear magnetic resonance
PC: phosphatidylcholine
PGS: polygenic score
PUFA: polyunsaturated fatty acid
QC: quality control
SHBG: sex hormone-binding globulin
SM: sphingomyelin

## References

[1] Stephenson DJ, Hoeferlin LA, Chalfant CE. Lipidomics in translational research and the clinical significance of lipid-based biomarkers. Transl Res. 2017;189:13–29. doi: 10.1016/j.trsl.2017.06.006 28668521 PMC5659874

[2] Butler LM, Perone Y, Dehairs J, Lupien LE, de Laat V, Talebi A, et al. Lipids and cancer: Emerging roles in pathogenesis, diagnosis and therapeutic intervention. Adv Drug Deliv Rev. 2020;159:245–293. doi: 10.1016/j.addr.2020.07.013 32711004 PMC7736102

[3] Schneider M, Levant B, Reichel M, Gulbins E, Kornhuber J, Müller CP. Lipids in psychiatric disorders and preventive medicine. Neurosci Biobehav Rev. 2017;76:336–362. doi: 10.1016/j.neubiorev.2016.06.002 27317860

[4] Yusuf S, Joseph P, Rangarajan S, Islam S, Mente A, Hystad P, et al. Modifiable risk factors, cardiovascular disease, and mortality in 155 722 individuals from 21 high-income, middle-income, and low-income countries (PURE): a prospective cohort study. Lancet. 2020;395:795–808. 31492503 10.1016/S0140-6736(19)32008-2 PMC8006904

[5] Torkamani A, Wineinger NE, Topol EJ. The personal and clinical utility of polygenic risk scores. Nat Rev Genet. 2018;19:581–590. doi: 10.1038/s41576-018-0018-x 29789686

[6] Mars N, Koskela JT, Ripatti P, Kiiskinen TTJ, Havulinna AS, Lindbohm JV, et al. Polygenic and clinical risk scores and their impact on age at onset and prediction of cardiometabolic diseases and common cancers. Nat Med. 2020;26:549–557. doi: 10.1038/s41591-020-0800-0 32273609

[7] Khera AV, Chaffin M, Aragam KG, Haas ME, Roselli C, Choi SH, et al. Genome-wide polygenic scores for common diseases identify individuals with risk equivalent to monogenic mutations. Nat Genet. 2018;50:1219. doi: 10.1038/s41588-018-0183-z 30104762 PMC6128408

[8] Quehenberger O, Armando AM, Brown AH, Milne SB, Myers DS, Merrill AH, et al. Lipidomics reveals a remarkable diversity of lipids in human plasma. J Lipid Res. 2010;51:3299–3305. doi: 10.1194/jlr.M009449 20671299 PMC2952570

[9] Han X. Lipidomics for studying metabolism. Nat Rev Endocrinol. 2016;12:668–679. doi: 10.1038/nrendo.2016.98 27469345

[10] Fang S, Holmes MV, Gaunt TR, Davey Smith G, Richardson TG. Constructing an atlas of associations between polygenic scores from across the human phenome and circulating metabolic biomarkers. Elife. 2022;11:e73951. doi: 10.7554/eLife.73951 36219204 PMC9553209

[11] Julkunen H, Cichońska A, Tiainen M, Koskela H, Nybo K, Mäkelä V, et al. Atlas of plasma NMR biomarkers for health and disease in 118,461 individuals from the UK Biobank. Nat Commun. 2023;14:604. doi: 10.1038/s41467-023-36231-7 36737450 PMC9898515

[12] Widén E, Junna N, Ruotsalainen S, Surakka I, Mars N, Ripatti P, et al. How Communicating Polygenic and Clinical Risk for Atherosclerotic Cardiovascular Disease Impacts Health Behavior: an Observational Follow-up Study. Circ Genom Precis Med. 2022;15:e003459. doi: 10.1161/CIRCGEN.121.003459 35130028

[13] Gerl MJ, Klose C, Surma MA, Fernandez C, Melander O, Männistö S, et al. Machine learning of human plasma lipidomes for obesity estimation in a large population cohort. PLoS Biol. 2019;17:e3000443. doi: 10.1371/journal.pbio.3000443 31626640 PMC6799887

[14] Yuan S, Li X, Morange PE, Bruzelius M, Larsson SC. On Behalf Of The Invent Consortium. Plasma Phospholipid Fatty Acids and Risk of Venous Thromboembolism: Mendelian Randomization Investigation. Nutrients. 2022;14:3354. doi: 10.3390/nu14163354 36014859 PMC9412533

[15] Martin S, Tyrrell J, Thomas EL, Bown MJ, Wood AR, Beaumont RN, et al. Disease consequences of higher adiposity uncoupled from its adverse metabolic effects using Mendelian randomisation. Elife. 2022;11:e72452. doi: 10.7554/eLife.72452 35074047 PMC8789289

[16] Huynh K, Barlow CK, Jayawardana KS, Weir JM, Mellett NA, Cinel M, et al. High-Throughput Plasma Lipidomics: Detailed Mapping of the Associations with Cardiometabolic Risk Factors. Cell Chem Biol. 2019;26:71–84.e4. doi: 10.1016/j.chembiol.2018.10.008 30415965

[17] Beyene HB, Olshansky G, Smith AAT, Giles C, Huynh K, Cinel M, et al. High-coverage plasma lipidomics reveals novel sex-specific lipidomic fingerprints of age and BMI: Evidence from two large population cohort studies. PLoS Biol. 2020;18:e3000870. doi: 10.1371/journal.pbio.3000870 32986697 PMC7544135

[18] Yin X, Willinger CM, Keefe J, Liu J, Fernández-Ortiz A, Ibáñez B, et al. Lipidomic profiling identifies signatures of metabolic risk. EBioMedicine. 2020;51:102520. doi: 10.1016/j.ebiom.2019.10.046 31877415 PMC6938899

[19] Razquin C, Toledo E, Clish CB, Ruiz-Canela M, Dennis C, Corella D, et al. Plasma Lipidomic Profiling and Risk of Type 2 Diabetes in the PREDIMED Trial. Diabetes Care. 2018;41:2617–2624. doi: 10.2337/dc18-0840 30327364 PMC6245212

[20] Fernandez C, Surma MA, Klose C, Gerl MJ, Ottosson F, Ericson U, et al. Plasma Lipidome and Prediction of Type 2 Diabetes in the Population-Based Malmö Diet and Cancer Cohort. Diabetes Care. 2020;43:366–373. 31818810 10.2337/dc19-1199

[21] Chen K, Zheng J, Shao C, Zhou Q, Yang J, Huang T, et al. Causal effects of genetically predicted type 2 diabetes mellitus on blood lipid profiles and concentration of particle-size-determined lipoprotein subclasses: A two-sample Mendelian randomization study. Front Cardiovasc Med. 2022;9:965995. doi: 10.3389/fcvm.2022.965995 36312274 PMC9606322

[22] Tamlander M, Mars N, Pirinen M, FinnGen, Widén E, Ripatti S. Integration of questionnaire-based risk factors improves polygenic risk scores for human coronary heart disease and type 2 diabetes. Commun Biol. 2022;5:158. doi: 10.1038/s42003-021-02996-0 35197564 PMC8866413

[23] Stellato RK, Feldman HA, Hamdy O, Horton ES, McKinlay JB. Testosterone, sex hormone-binding globulin, and the development of type 2 diabetes in middle-aged men: prospective results from the Massachusetts male aging study. Diabetes Care. 2000;23:490–494. doi: 10.2337/diacare.23.4.490 10857940

[24] Ding EL, Song Y, Manson JE, Hunter DJ, Lee CC, Rifai N, et al. Sex hormone-binding globulin and risk of type 2 diabetes in women and men. N Engl J Med. 2009;361:1152–1163. doi: 10.1056/NEJMoa0804381 19657112 PMC2774225

[25] Wang Q, Kangas AJ, Soininen P, Tiainen M, Tynkkynen T, Puukka K, et al. Sex hormone-binding globulin associations with circulating lipids and metabolites and the risk for type 2 diabetes: observational and causal effect estimates. Int J Epidemiol. 2015;44:623–637. doi: 10.1093/ije/dyv093 26050255

[26] Pitteloud N, Mootha VK, Dwyer AA, Hardin M, Lee H, Eriksson KF, et al. Relationship between testosterone levels, insulin sensitivity, and mitochondrial function in men. Diabetes Care. 2005;28:1636–1642. doi: 10.2337/diacare.28.7.1636 15983313

[27] Rao PM, Kelly DM, Jones TH. Testosterone and insulin resistance in the metabolic syndrome and T2DM in men. Nat Rev Endocrinol. 2013;9:479–493. doi: 10.1038/nrendo.2013.122 23797822

[28] Ottarsdottir K, Nilsson AG, Hellgren M, Lindblad U, Daka B. The association between serum testosterone and insulin resistance: a longitudinal study. Endocr Connect. 2018;7:1491–1500. doi: 10.1530/EC-18-0480 30592706 PMC6311464

[29] Martin S, Cule M, Basty N, Tyrrell J, Beaumont RN, Wood AR, et al. Genetic Evidence for Different Adiposity Phenotypes and Their Opposing Influences on Ectopic Fat and Risk of Cardiometabolic Disease. Diabetes. 2021;70:1843–1856. doi: 10.2337/db21-0129 33980691

[30] Yaghootkar H, Lotta LA, Tyrrell J, Smit RA, Jones SE, Donnelly L, et al. Genetic Evidence for a Link Between Favorable Adiposity and Lower Risk of Type 2 Diabetes, Hypertension, and Heart Disease. Diabetes. 2016;65:2448–2460. doi: 10.2337/db15-1671 27207519 PMC5386140

[31] Mars N, Kerminen S, Feng YA, Kanai M, Läll K, Thomas LF, et al. Genome-wide risk prediction of common diseases across ancestries in one million people. Cell Genom. 2022;2:None. doi: 10.1016/j.xgen.2022.100118 35591975 PMC9010308

[32] Tabassum R, Ruotsalainen S, Ottensmann L, Gerl MJ, Klose C, Tukiainen T, et al. Lipidome- and Genome-Wide Study to Understand Sex Differences in Circulatory Lipids. J Am Heart Assoc. 2022;11:e027103. doi: 10.1161/JAHA.122.027103 36193934 PMC9673737

[33] Surma MA, Herzog R, Vasilj A, Klose C, Christinat N, Morin-Rivron D, et al. An automated shotgun lipidomics platform for high throughput, comprehensive, and quantitative analysis of blood plasma intact lipids. Eur J Lipid Sci Technol. 2015;117:1540–1549. doi: 10.1002/ejlt.201500145 26494980 PMC4606567

[34] Matyash V, Liebisch G, Kurzchalia TV, Shevchenko A, Schwudke D. Lipid extraction by methyl-tert-butyl ether for high-throughput lipidomics. J Lipid Res. 2008;49:1137–1146. doi: 10.1194/jlr.D700041-JLR200 18281723 PMC2311442

[35] McDonald JG, Ejsing CS, Kopczynski D, Holčapek M, Aoki J, Arita M, et al. Introducing the Lipidomics Minimal Reporting Checklist. Nat Metab. 2022;4:1086–1088. doi: 10.1038/s42255-022-00628-3 35934691

[36] Herzog R, Schwudke D, Schuhmann K, Sampaio JL, Bornstein SR, Schroeder M, et al. A novel informatics concept for high-throughput shotgun lipidomics based on the molecular fragmentation query language. Genome Biol. 2011;12:R8. doi: 10.1186/gb-2011-12-1-r8 21247462 PMC3091306

[37] Herzog R, Schuhmann K, Schwudke D, Sampaio JL, Bornstein SR, Schroeder M, et al. Lipidxplorer: A software for consensual cross-platform lipidomics. PLoS ONE. 2012;7:e29851. doi: 10.1371/journal.pone.0029851 22272252 PMC3260173

[38] Loh PR, Danecek P, Palamara PF, Fuchsberger C, Reshef YA, Finucane HK, et al. Reference-based phasing using the Haplotype Reference Consortium panel. Nat Genet. 2016;48:1443–1448. doi: 10.1038/ng.3679 27694958 PMC5096458

[39] Browning BL, Browning SR. Genotype imputation with millions of reference samples. Am J Hum Genet. 2016;98:116–126. doi: 10.1016/j.ajhg.2015.11.020 26748515 PMC4716681

[40] Kurki MI, Karjalainen J, Palta P, Sipilä TP, Kristiansson K, Donner KM, et al. FinnGen provides genetic insights from a well-phenotyped isolated population. Nature. 2023;613:508–518. doi: 10.1038/s41586-022-05473-8 36653562 PMC9849126

[41] Ge T, Chen CY, Ni Y, Feng YA, Smoller JW. Polygenic prediction via Bayesian regression and continuous shrinkage priors. Nat Commun. 2019;10:1776. doi: 10.1038/s41467-019-09718-5 30992449 PMC6467998

[42] Purcell S, Neale B, Todd-Brown K, Thomas L, Ferreira MA, Bender D, et al. PLINK: a tool set for whole-genome association and population-based linkage analyses. Am J Hum Genet. 2007;81: 559–575. doi: 10.1086/519795 17701901 PMC1950838

