# Supplementary figures and images for: Polygenic scores for complex traits are associated with changes in concentration of circulating lipid species
Source: PLoS Biol. 2024 Sep 26;22(9):e3002830. doi: 10.1371/journal.pbio.3002830 (PMC11460696; doi:10.1371/journal.pbio.3002830)

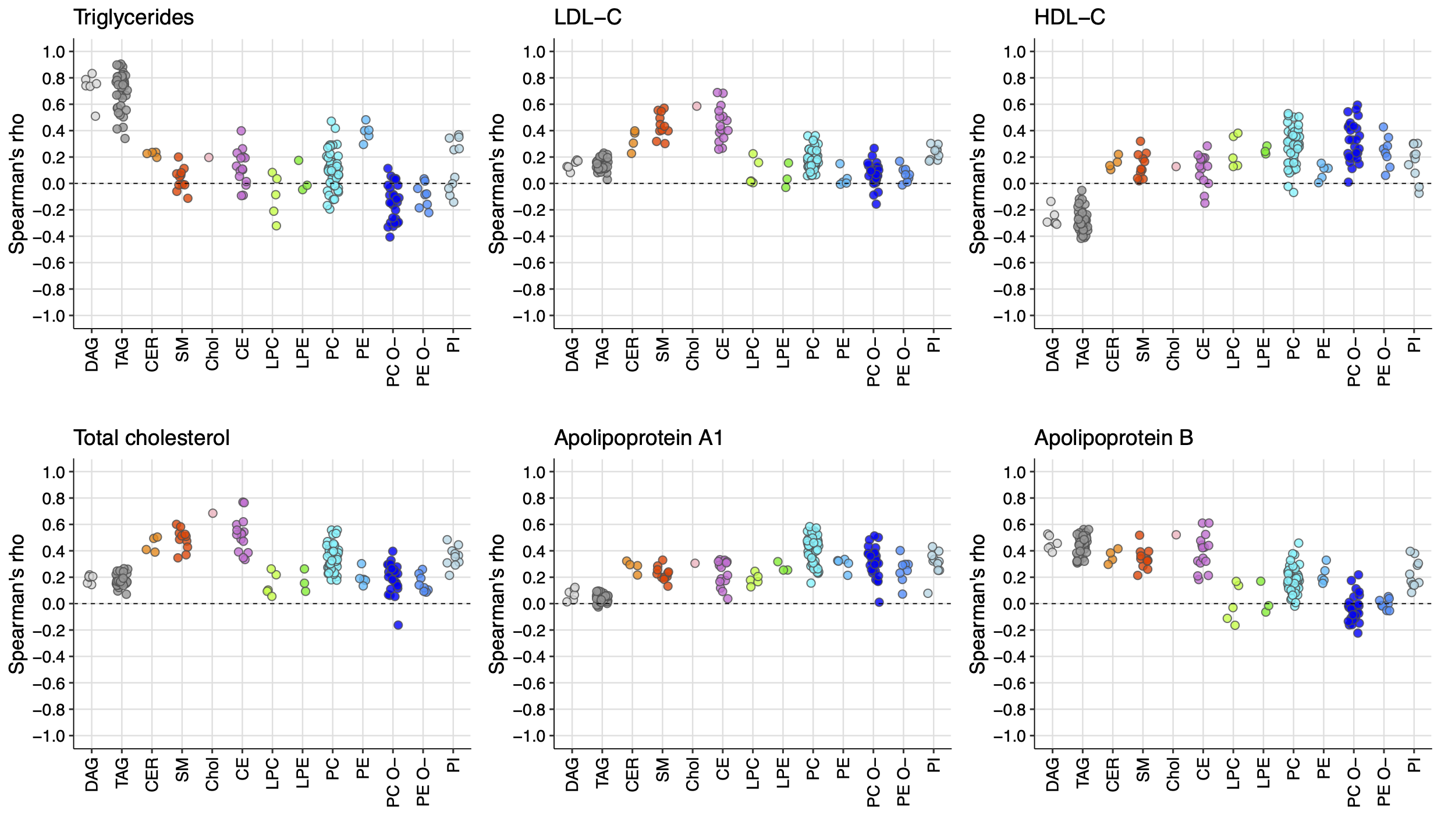

Supplement: S1 Fig — Each point in the plots represents the Spearman correlation coefficient (rho). The lipid species are grouped and colored by the lipid classes they belong. The data underlying this figure may be found in S1 Data. (TIFF) [file pbio.3002830.s017.tiff]

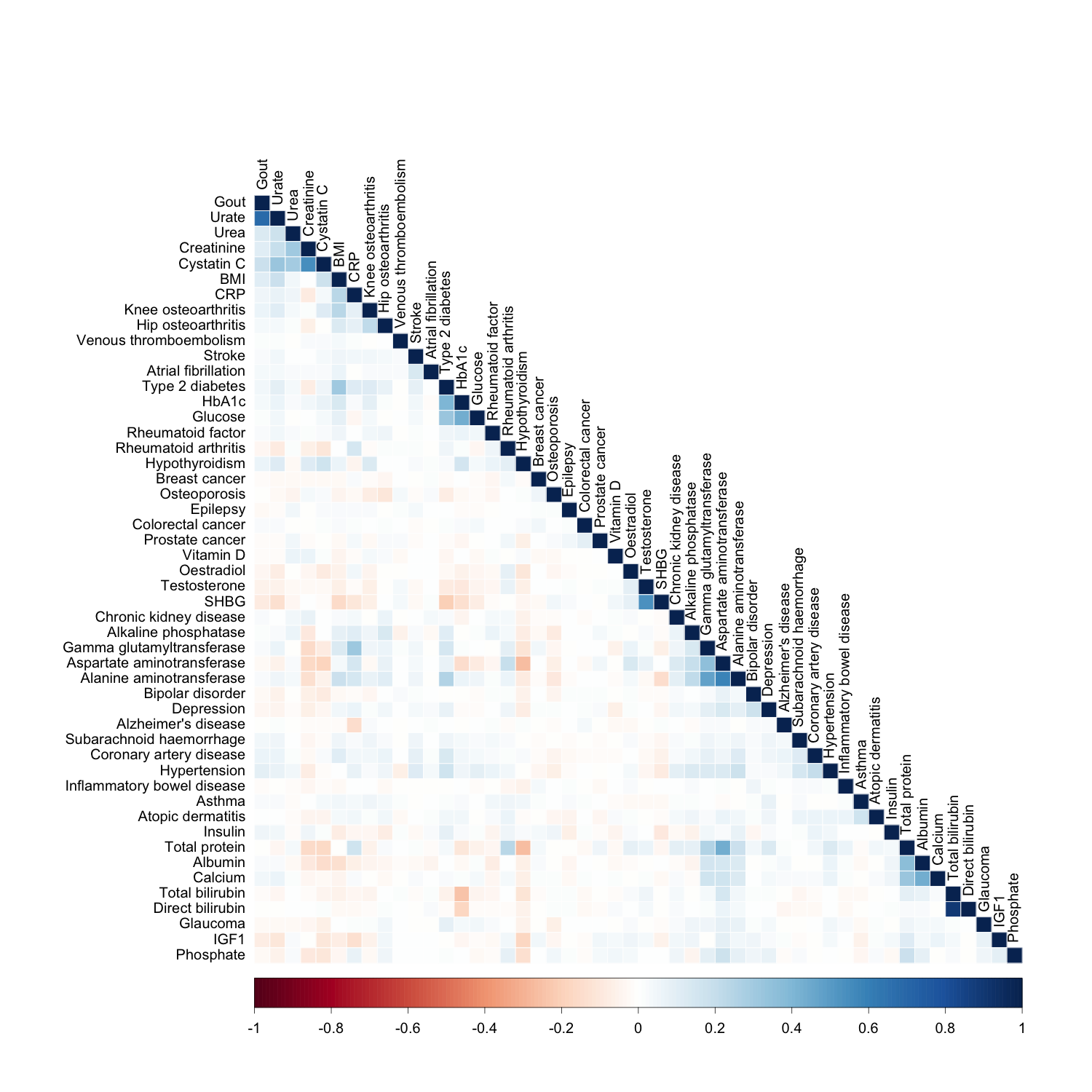

Supplement: S2 Fig — Pair-wise Spearman correlation between the PGS for all 50 complex traits included in the study are shown in the heatmap. The data underlying this figure may be found in S1 Data. (TIFF) [file pbio.3002830.s018.tiff]

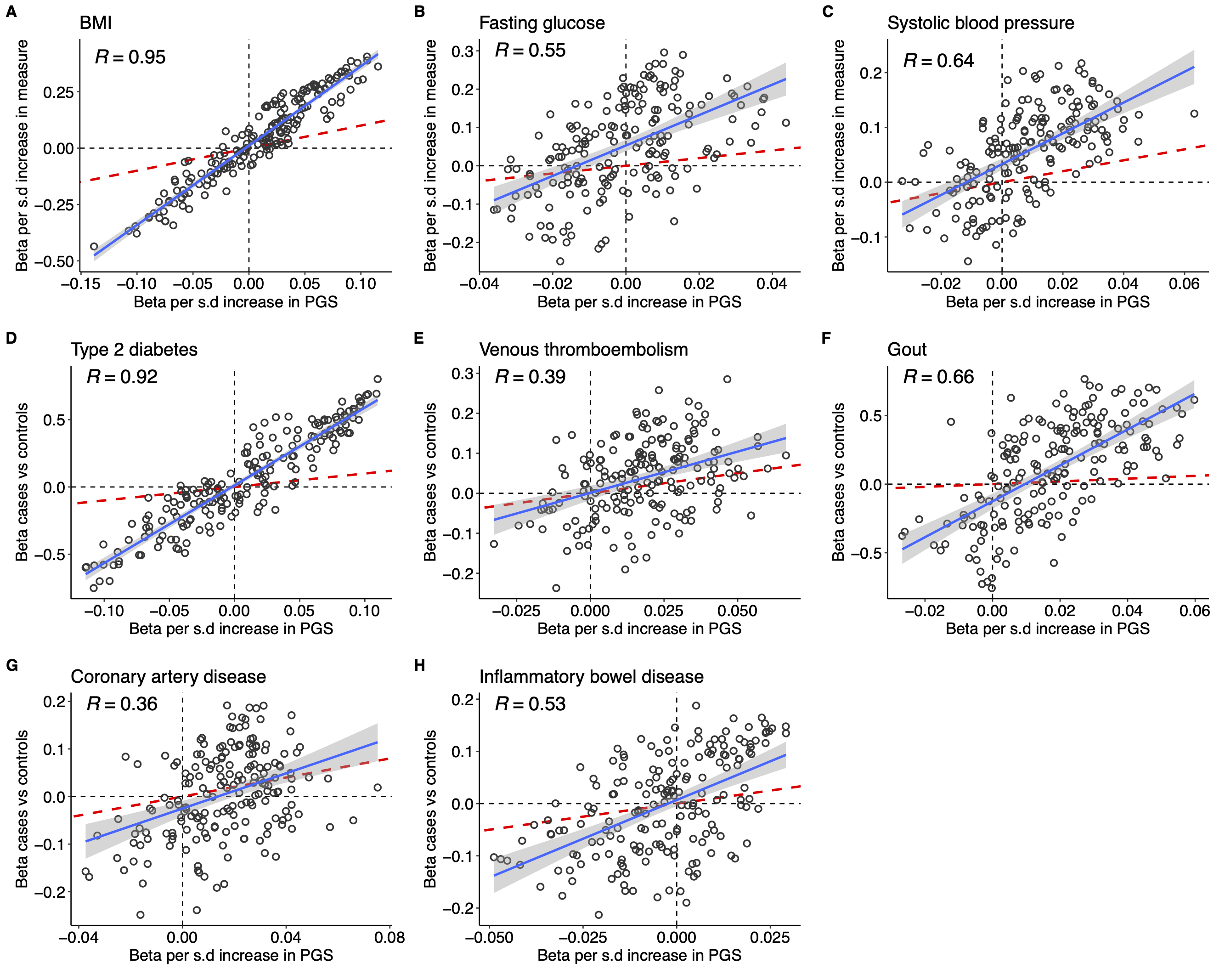

Supplement: S3 Fig — Scatter plots show correlations between the effect sizes (beta per SD increase in PGS) obtained from the linear regression analyses for association of the PGS with the lipids on x-axis and the corresponding effect sizes for the association of actual measure or disease status with the lipids on y-axis. R represents the Spearman correlation coefficient. The red line represents the regression line with slope of one and intercept zero, while blue line represents the regression line of the model. The data underlying this figure may be found in S1 Data. (TIFF) [file pbio.3002830.s019.tiff]

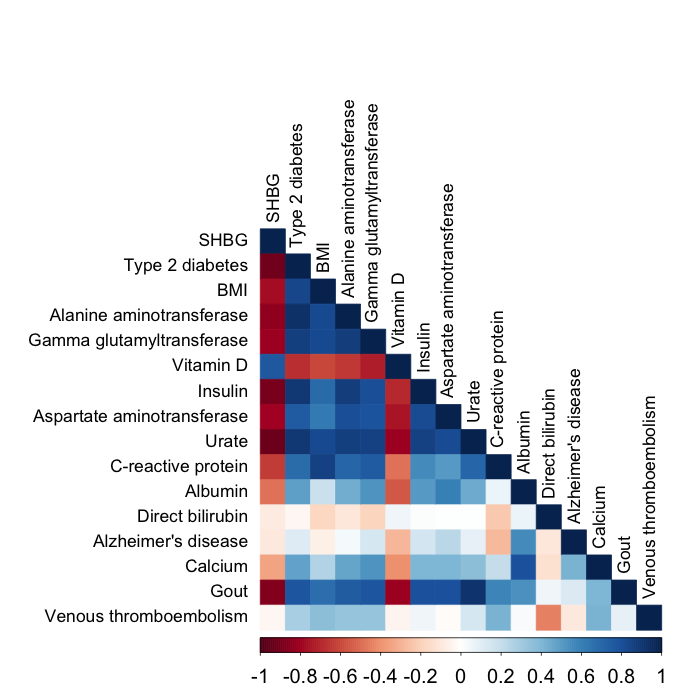

Supplement: S4 Fig — Pair-wise Spearman correlation between the effect sizes of each pair of PGSs for lipid species are shown in the heatmap. The z-scores (beta/SD) were used to calculate the correlations between the PGSs. Only the PGSs with at least 10 significant PGS-lipid associations are plotted. The data underlying this figure may be found in S1 Data. (TIFF) [file pbio.3002830.s020.tiff]

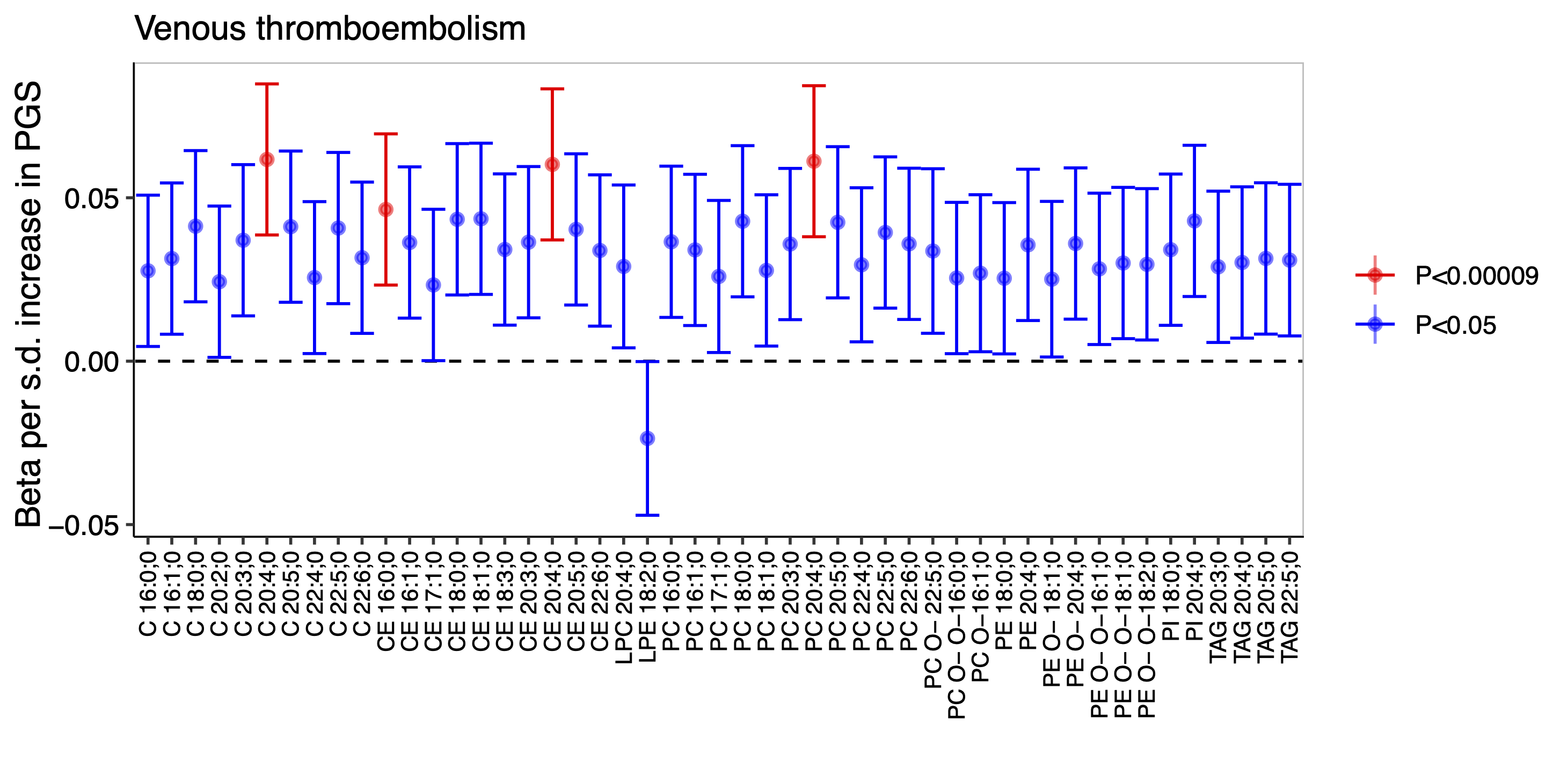

Supplement: S5 Fig — Fatty acid aggregates were calculated across all lipid classes (e.g., C 16:0;0), whereas fatty acid aggregates per lipid class were determined as the sum of the molar concentrations of fatty residues within each lipid class (e.g., PC 16:0;0). Only the lipids with P < 0.05 are plotted. The data underlying this figure may be found in S1 Data. (TIFF) [file pbio.3002830.s021.tiff]
